# Supplementary material for: Blood Donor RBC Genotyping at the National Advisory Unit on Immunohematology at Oslo University Hospital
Source: Biomedicines. 2025 Nov 27;13(12):2907. doi: 10.3390/biomedicines13122907 (PMC12731193; doi:10.3390/biomedicines13122907)
Supplement: Supplementary file 1 [file biomedicines-13-02907-s001.zip › biomedicines-3879233-Supplementary Materials.pdf]

Supplementary Materials

Probabilistic estimation of Rh haplotype frequencies

This supplementary section presents the probabilistic estimation of Rh haplotype frequencies in the donor cohort, performed to assess the internal consistency of RhD–C–E phenotypes and validate the representativeness of the study population.

The analysis was based on combined serologic RhD typing and RHCE genotyping (C and E) data from all 2,501 donors.

Frequencies of the major Rh haplotypes — R^1 (DCe), R^2 (DcE), R^0 (Dce), R^z (DCE), r (dce), r′ (dCe), and r″ (dcE) — were estimated using Microsoft Excel Solver (GRG Nonlinear algorithm) under Hardy–Weinberg equilibrium assumptions.

The optimization minimized the sum of squared errors (SSE) between observed and modelled group proportions, with all frequencies constrained to be non-negative and to sum to one.

The Solver converged rapidly to a stable solution (SSE = 0.0086), confirming an excellent model fit.

Confidence intervals (95%) were calculated using binomial approximations:

$$f \pm 1.96\sqrt{\frac{f\,(1-f)}{(2N)}}, \text{where } N = 2{,}501.$$

Supplementary Table S1.

Estimated Rh haplotype frequencies in blood donors (N = 2,501; 2N = 5,002 haplotypes).

Haplotype	Frequency (%)	95% CI (%)	Expected phenotype(s)	Observed group(s)	Modelled group(s)	Notes
R^1 (DCe)	38.9	37.5–40.3	D^+ C^+ E^-	g1	g1	Main D^+ C^+ E^- haplotype
R^2 (DcE)	9.8	9.0–10.6	D^+ C^- E^+	g2	g2	Moderate frequency
R^0 (Dce)	2.3	1.9–2.7	D^+ C^- E^-	g3	g3	Low-frequency D^+ haplotype
R^z (DCE)	0.0	0.0–0.0	D^+ C^+ E^+	g7	g7	Very rare
r (dce)	49.0	47.6–50.4	D^- C^- E^-	g4	g4	Predominant D^- haplotype
r′ (dCe)	0.0	0.0–0.0	D^- C^+ E^-	g5	g5	Rare
r″ (dcE)	0.0	0.0–0.0	D^- C^- E^+	g6	g6	Rare

Model fit: SSE = 0.0086.

Method: Solver (GRG Nonlinear) minimizing squared deviations between observed and modelled RhD–C–E group proportions under Hardy–Weinberg equilibrium.

Supplementary Table S2.

Observed and modelled proportions of RhD–C–E phenotype groups (N = 2 501)

Group	RhD–C–E phenotype	Observed (%)	Modelled (%)	Absolute residual Δ (%)
g1	$D^{+}\ C^{+}\ E^{-}$	56.7	55.0	1.7
g2	$D^{+}\ C^{-}\ E^{+}$	16.8	11.0	5.8
g3	$D^{+}\ C^{-}\ E^{-}$	1.6	2.3	0.7
g4	$D^{-}\ C^{-}\ E^{-}$	23.3	24.0	0.7
g5	$D^{-}\ C^{+}\ E^{-}$	0.4	0.0	0.4
g6	$D^{-}\ C^{-}\ E^{+}$	0.5	0.0	0.5
g7	$D^{+}\ C^{+}\ E^{+}$	0.7	7.6	7.0
g8	$D^{-}\ C^{+}\ E^{+}$	0.1	0.0	0.1
**Σ**		**100.0**	**100.0**	

Supplementary Figure S1. Observed vs. modelled RhD–C–E group proportions

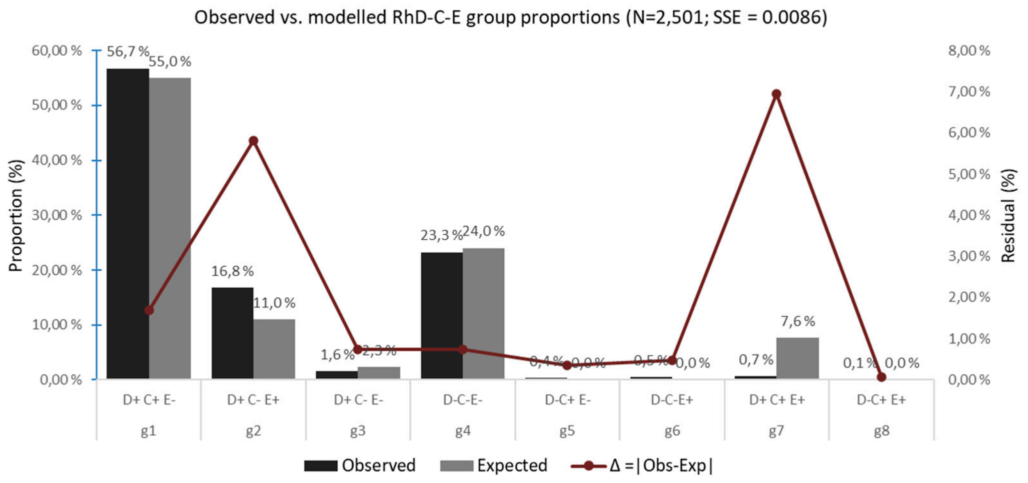

Observed vs. modelled RhD–C–E group proportions

Observed (dark) and modelled (light) proportions for each group (g1–g8).
The overlayed line shows absolute residuals (Δ = |Observed − Expected|).
Model fit was excellent (SSE = 0.0086), and all residuals were <0.07.

Supplementary File S1 (Excel)

File: Rh_Haplotypes_Probabilistic_Modeling.xlsx

Description:
Contains the full dataset and calculations for the probabilistic Rh haplotype analysis.
Sheet *HaplotypeFit* includes observed and modelled group proportions used for Solver optimization.
CI_calculations provides 95% confidence intervals for haplotype frequencies.
Observed_vs_Expected summarizes model fit and residuals, and SummaryTable_S2 reproduces the table above.
This file enables full reproducibility of the probabilistic estimation.
